# Role of professional networks on social media in addressing clinical questions at general practice: a cross-sectional study of general practitioners in Australia and New Zealand

**DOI:** 10.1186/s12875-019-0931-x

**Published:** 2019-03-06

**Authors:** Loai Albarqouni, Tammy Hoffmann, Katrina McLean, Karen Price, Paul Glasziou

**Affiliations:** 1 Centre for Research in Evidence Based Practice, Bond University, 14 University Dr, Robina, QLD 4229 Australia; 2 Faculty of Health Science and Medicine, Bond University, 14 University Dr, Robina, QLD 4229 Australia; 3 School of Primary Health Care, Monash University, Melbourne, Australia

**Keywords:** Information needs, Clinical questions, Social media networks, Evidence-based practice

## Abstract

**Background:**

Clinicians frequently have questions about patient care. However, for more than half of the generated questions, answers are never pursued, and if they are, often not answered satisfactorily. We aimed to characterise the clinical questions asked and answers provided by general practitioners (GP) through posts to a popular professional social media network.

**Methods:**

In this cross-sectional study, we analysed clinical questions and answers posted between January 20th and February 10th 2018 on a popular GP-restricted (Australia, New Zealand) Facebook group. Each clinical question was categorised according to ‘background’ or ‘foreground’ question; type (e.g. treatment, diagnosis); and the clinical topic (e.g. cardiovascular). Each answer provided in response to included questions was categorised into: (i) short answer (e.g. agree/disagree); (ii) provided an explanation to justify the answer; and (iii) referred to a published relevant evidence resource.

**Results:**

Of 1060 new posts during the study period, 204 (19%) included a clinical question. GPs most commonly asked about treatment (*n* = 87; 43%) and diagnosis (*n* = 59; 29%). Five major topics (23% skin, 10% psychology, 9% cardiovascular, 8% female genital, and 7% musculoskeletal) accounted for 118 (58%) questions. Each question received on average 10 (SD = 9) answers: 42% were short; 51% provided an explanation; and only 6% referred to relevant research evidence. Only 3 answers referred to systematic reviews.

**Conclusions:**

In this sample of Australian and New Zealand GPs, who were members of a GP social media group, GPs asked clinical questions that can be organised into a limited number of question types and topics. This might help guide the development of GP learning programs.

## Background

There has been a rapid expansion of information in health care over the last few decades [1]. The challenge of keeping up with this information overload in health care is becoming harder, if not impossible [2, 3]. An information paradox exists, as despite being overwhelmed by this huge amount of information, clinicians frequently face personal knowledge gaps, ask clinical questions about patient care, and have many unanswered questions [1, 4].

A systematic review of clinical questions raised by clinicians showed that clinicians ask about 1 question every 2 patients [5]. However, for more than half of the generated questions, answers are never pursued, and if they are, often not answered satisfactorily [5, 6] – suggested missed opportunities for continuous learning. Lack of time and clinicians’ doubt about the existence and usefulness of relevant answers are the most commonly reported barriers to pursuing the answers for their clinical questions [5, 7]. Thus, addressing clinicians’ personal knowledge gaps provides an opportunity for continuing learning, and enhanced patient care. This is especially important for general practitioners (GPs) as their information needs are much broader than that of other specialties because of the wider spectrum of clinical problems encountered [8]. Consulting colleagues to answer clinical questions is one of the most common strategies that clinicians adopt to cope with the information overload [1, 2]. Clinicians are increasingly using social media to communicate and network with colleagues, share information, and disseminate research findings [9]. Thus, understanding clinicians’ use of social media networks to overcome information overload and address clinical questions generated from patient care is warranted. We aimed to characterise the clinical questions asked and answers provided by general practitioners and posted to a popular professional social media network.

## Methods

In this cross-sectional study, we analysed all clinical questions posted on a popular GP-restricted Facebook group ‘GPs Down Under’ between January 20th and February 10th 2018. ‘GPs Down Under’ is a GP community-led closed professional Facebook group restricted to GPs practising in Australia and New Zealand. It has over 5800 members and generates over 50 posts per day.

The criteria for GPDU group membership include being a GP or a GP registrar and working in general practice with registration to practice in Australia and/or New Zealand. A three-step verification procedure is used. Two of the co-authors (KM and KP) were co-developers and are administrators of the GPDU Facebook group.

Two of the co-authors (KM and KP) scraped all the data (including each original post and all subsequent comments and replies to that post) of the posts that were posted during the study period. One of the authors who is also a member of GPDU (PG) de-identified the data and developed a de-identified anonymised dataset for screening and analysis.

We screened all posts that were posted to the group during the study period to identify those that included a clinical question (as defined by Ely et al. [10] - ‘questions about medical knowledge that could potentially be answered by general sources such as textbooks and journals, not questions about patient data that would be answered by the medical record’) – the focus of this analysis is on clinical questions posts. We categorised each included question as ‘background’ (e.g. What is myocardial infarction?) or ‘foreground’ question (e.g. In adult patients with myocardial infarction, does aspirin increase survival?). We also classified the type of each question (e.g. treatment, diagnosis) per the taxonomy used by Ely et al. [10]. We also classified the clinical topics of each included question according to the revised version of International Classification of Primary Care (ICPC-2) [11]. ICPC is a coding system co-developed and endorsed by the World Organization of Family Doctors to allow for more appropriate classification of data frequently encountered in a primary care setting [12, 13]. We screened all comments for answers provided in response to each question and classified each answer as: (i) short answer (e.g. yes/no or agree/disagree); (ii) provided an explanation (e.g. justify the answer or provide supporting clinical examples); and (iii) referred to a published relevant evidence resource (e.g. provided a website link to a research article or guideline). Three of the authors (LA, TH, and PG) independently analysed a random sample of 5% of posts and continued discussion until consensus was attained. LA coded the included questions and answers of the rest of included posts. Any uncertainties in the coding decisions were resolved by one of the co-authors with extensive experience in primary care (PG).

The study was approved by Bond University Human Research Ethics Committee. Group members were informed that all new posts during the study period would be anonymously used for research purposes without breaching members’ privacy.

## Results

During the study period, 504 GPs contributed a total of 1060 new posts, of which 204 (19%) included a clinical question. Of these 204 included questions, 174 (85%) were foreground and 30 (15%) background questions. The characteristics of clinical questions posted to GPDU group are presented in Table 1. Overall, most asked questions (165; 81%) concerned around 14 (30%) of the 42 identified question types: 87 (43%) about treatment followed by 59 (29%) diagnosis. The most frequently asked question types were: (i) 34 (17%) questions about the efficacy or the indication of a treatment (e.g. Does procedure/treatment x work for condition y?); (ii) 28 (14%) questions about the management (i.e. diagnostic or therapeutic) of a condition or finding (e.g. How should I manage condition/finding/situation y?); (iii) 23 (11%) questions about the cause or the interpretation of unspecified multiple findings (e.g. What does this patient have given these findings?). Table 2 lists the 10 most frequently asked clinical question types, with examples from the included questions.

**Table 1:** Characteristics of clinical questions posted to the GPDU group (*n* = 204)

	N (%)
Total clinical questions	204
Background Questions	30 (15%)
Foreground Questions	174 (85%)
Reaction to each question	
Comments (number of comments per question; median [IQR])	15 [7–28]
Answers (number of answers per question; median [IQR])	7 [4–14]
Short answers (% of all answers per question)	42%
With an explanation (% of all answers per question)	51%
Referred to published resources (% of all answers per question)	6%
Referred to a systematic review (number of answers of all answers)	3

**Table 2:** The most frequently asked clinical questions’ types with examples from the included questions

Question Type	Description	No (%)	Example
2.2.1.1 Treatment Efficacy or indications of a treatment (but not limited to drug treatment)	How should I treat finding/condition y (given situation z)? What is the efficacy of treatment/procedure x (for condition y)?	34 (16.7%)	What are the treatment options for a second therapy for *H. pylori* infection after a failure with Nexium Hp7?
3.1.1.1 Management Management of a condition or finding (not specifying diagnostic or therapeutic management)	How should I manage condition/finding/situation y? What management options are there in situation y?	28 (13.7%)	How should I manage ilioinguinal nerve entrapment?
1.1.4.1 Diagnosis Cause or interpretation of unspecified or multiple clinical findings	Could this patient have condition y given these findings? What is the differential diagnosis of these findings? What is the likelihood that this patient has condition y given these findings?	23 (11.3%)	Middle-age diabetic lady with osteoarthritis and chronic skin changes. She presented with a recent papules (non-itchy, rough, and translucent). No history of insect bites, new drugs. What is the differential diagnosis for her condition?
2.1.2.1 Treatment Efficacy or indications of a drug or drug of choice	Is drug x indicated in situation y or for condition y? What are the indications for drug x? What is the drug of choice for condition y?	16 (7.8%)	Is Liraglutide indicated in a female non-diabetic patient with BMI of 38?
3.2.1.1 Management Practices of other providers	How do other providers manage condition y? Why did provider x treat the patient this way?	11 (5.4%)	How many of you prescribe dietary modification as first line management of mild hypertension? If yes, do you give general or specific dietary advice?
1.3.1.1 Diagnosis Indication or efficacy of a test (e.g. lab, imaging, physical exam)	Is test x indicated in situation y? What is the best test in situation y?	10 (4.9%)	Is joint aspiration for every suspected case of recurrent gout is needed to confirm the diagnosis if considering long term urate-lowering therapy?
1.3.3.1 Diagnosis Accuracy of a test (e.g. lab, imaging, physical exam)	How good is test x in situation y? What are the performance characteristics (sensitivity, specificity, etc.) of test x in situation y?	8 (3.9%)	How good is Bone scan compared to CT scane in detecting a suspected stress fracture in the foot with a normal X-ray.
2.1.3.1 Treatment Adverse effects of a treatment	Are these findings can be caused by a drug or adverse effects of drug?	7 (3.4%)	A patients with rheumatoid arthritis on adalimumab, presented with a tendon rupture. Could this be a side effect of adalimumab?
2.1.3.3 Treatment Contraindications of a treatment	What are the safety issues or contraindications of treatment x (includes pregnancy and breast feeding)?	5 (2.5%)	Middle-age diabetic female patient on Metformin with mild microalbuminuria and normal creatinine. Is NSAIDs contraindicated in this patients due to renal complications?
1.1.1.1 Diagnosis Cause or interpretation of specified clinical findings (e.g. symptoms)	What is the cause of symptom x? Could symptom x be condition y or be a result of condition y?	5 (2.5%)	Asymptomatic middle-age lady presented with acute-onset of midline painless lump in the palate. What is the cause of this? Could be mucinous cyst?

The clinical question topics were fairly distributed across all the clinical topics reflecting the range of patients seen by GPs. However, over half of all included clinical questions (*n* = 118; 58%) concerned five major clinical topics. The five most frequently addressed topics were skin (*n* = 47; 23%, 11 about skin neoplasm/lesion and 9 were related to a ‘rash’), mental health (*n* = 20; 10%), cardiovascular (*n* = 19; 9%), women’s health (*n* = 17; 8%), and musculoskeletal (*n* = 15; 7%). Table 3 shows the distribution of clinical questions across the clinical topics.

**Table 3:** The distribution of clinical questions across clinical topics per ICPC-2 classification system

Clinical Topic	No (%)
Skin	47 (23%)
Psychological	20 (9.8%)
Cardiovascular	19 (9.3%)
Female Genital	17 (8.3%)
Musculoskeletal	15 (7.4%)
Neurological	14 (6.9%)
Digestive	13 (6.4%)
Pregnancy, Childbearing, Family Planning	12 (5.9%)
Endocrine/Metabolic and Nutritional	12 (5.9%)
General and Unspecified	10 (4.9%)
Respiratory	6 (2.9%)
Blood, Blood Forming Organs and Immune Mechanism	5 (2.5%)
Eye	4 (2%)
Urological	3 (1.5%)
Male Genital	3 (1.5%)
Social Problems	2 (1%)
Ear	1 (0.5%)

The 204 included questions elicited 4065 comments, with a mean of 20 (SD 19) comments per included question (i.e. this refers to all comments that were posted as a reply to a clinical question; whether they provide an answer or not). GPDU members commented and provided answers for all 204 included questions. On average, 10 (SD 9) of the 20 (SD 19) comments were answers to the posted question; the remaining comments did not answer the clinical question originated in the post. On average, 42% (SD 27%) of these answers were short answers; 51% (SD 27%) were answers which provided an explanation or justification to the answer; and 6% (SD 11%) referred to published relevant evidence resource. Only three answers referred to evidence derived from systematic reviews (Table 1).

**Fig. 1:**
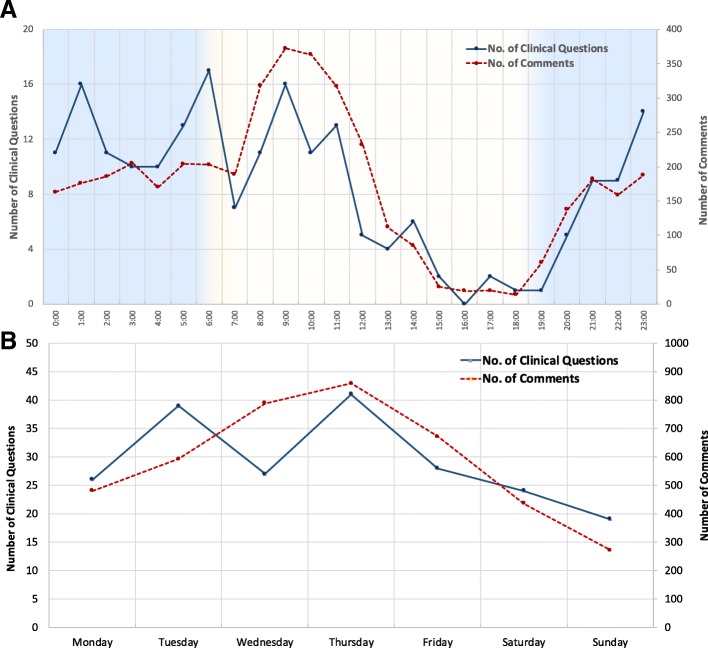
The activity of GPDU members in posting clinical questions (solid line) and comments (dashed line) per time (panel **a**) and day (panel **b**)

Figure 1 shows that engagement of GPDU members in asking and answering clinical questions per day and time. The engagement is peaked in the mornings (9 am) and on Thursdays, with a decline in the activity in late afternoon (4-5 pm) and on weekends.

## Discussions

In this study of GPs’ use of social media networks to answer their clinical questions – GPs posted approximately 10 questions per day. The majority of questions asked were about treatment and diagnosis and more than half of all included clinical questions were about a small number of clinical topics.

Our results regarding the question types are consistent with the results of a systematic review of 11 studies which examined 7012 clinical questions raised by clinicians (mostly GPs) at the point of care and found that the majority of clinical questions concerned treatment (34%) and diagnosis (24%) - with 30% of the question types accounting for 80% of the questions asked [5]. Similar, treatment and diagnosis were the most frequently observed types of clinical questions by Allan et al. (observed 38 GPs during 420 consultations) [14] and Green et al. (interviewed 64 residents after 401 consultations) [15].

Despite the wide spectrum of clinical presentations seen by GPs in practice, we found that most of the clinical questions asked about a handful of clinical topics. This is consistent with frequencies in previous studies of the most frequently asked clinical questions’ topics [14, 16], and most commonly managed conditions in general practice settings [11]. For instance, Bjerre et al. analysed 1871 questions asked by 88 Canadian GPs and found that musculoskeletal, skin, and cardiac were among the five most frequently asked question topics [17].

In this sample of GPs evidence-based resources (e.g. systematic reviews) were infrequently used to support answers to the posted clinical questions. This aligns with the findings of a systematic review of 19 studies that described information seeking behaviour of clinicians and found that evidence-based resources were rarely used by clinicians as a primary source of information to guide their decisions [18, 19].

A limitation of this study is that we focused on questions that GPs pursued, articulated, and posted a clinical question to find an answer (i.e. known unknowns), but we likely missed their unpursued recognised questions as well as their unrecognised questions (i.e. unknown unknowns). Direct observation studies and post-consultation interviews may better capture the information needs of clinicians at point of care (i.e. less susceptible to memory bias), although these methods might generate superfluous questions from clinicians because they are being observed or interviewed [7, 16]. Nor would they be useful in investigating the role of social media networks in addressing clinical questions asked by clinicians. Another limitation is that screening and coding of the posts were performed by one author, and three authors independently coded data from only a random sample of 5% of posts. Further, we analysed questions posted in a single restricted Facebook group by GPs who thought to be active social media users (504 GPs out of 5800 GPDU members), therefore, our findings may not be generalised to GPs who do not actively use social media or use other social media platform, or do not use social media at all. We also did not verify the validity of provided answers or the evidence used to support these answers. Thus, answers that referred to sources of evidence might not be accurate or correct and answers that did not cite a source of evidence might be evidence-based answers or correct (i.e. the lack of referral to evidence sources did not necessarily mean that these answers are not evidence-based).

Our findings that the majority of questions asked were about a limited number of questions types and topics suggest that questions raised on social media networks may be helpful in guiding the development of GP future continuous learning programs (e.g. tailored according to identified information needs) and research activities (e.g. by identifying research-practice evidence gaps) [20]. Although professional social media networks might be useful in providing evidence-based answers to clinical questions, the quality of the evidence underpinning the answers provided in social media should be questioned. Disadvantages of using the social media network in answering clinical questions might include: (i) GP members are responsible for discerning relevant answers and ascertaining the validity of the answers provided; and (ii) the possibility of delivering and perpetuation of unsound answers to a large group of GPs. Therefore, methods to enhance active dissemination of question-specific evidence-based information (such as by Facebook group administrators or evidence champions) are warranted [21].

## Conclusions

In this sample of Australian and New Zealand GPs, who were members of a GP social media group, the majority of clinical questions asked were about a limited number of questions types and topics which may help inform the development of GP future continuous learning programs and research activities. The validity of the evidence underpinning the answers provided for clinical questions asked in social media needs to be considered.

## Abbreviations

GP: General Practitioner
GPDU: General Practitioners Down Under
ICPC: International Classification of Primary Care
SD: Standard Deviation
